# Novel Treatment Strategies Targeting Myelin and Oligodendrocyte Dysfunction in Schizophrenia

**DOI:** 10.3389/fpsyt.2020.00379

**Published:** 2020-04-30

**Authors:** Danielle Gouvêa-Junqueira, Ana Caroline Brambilla Falvella, André Saraiva Leão Marcelo Antunes, Gabriela Seabra, Caroline Brandão-Teles, Daniel Martins-de-Souza, Fernanda Crunfli

**Affiliations:** ^1^Laboratory of Neuroproteomics, Department of Biochemistry and Tissue Biology, Institute of Biology, University of Campinas, Campinas, Brazil; ^2^Experimental Medicine Research Cluster (EMRC), University of Campinas, Campinas, Brazil; ^3^Instituto Nacional de Biomarcadores em Neuropsiquiatria, Conselho Nacional de Desenvolvimento Científico e Tecnológico, São Paulo, Brazil; ^4^D′Or Institute for Research and Education (IDOR), São Paulo, Brazil

**Keywords:** schizophrenia, oligodendrocyte, myelin, antipsychotic, glutamate

## Abstract

Oligodendrocytes are the glial cells responsible for the formation of the myelin sheath around axons. During neurodevelopment, oligodendrocytes undergo maturation and differentiation, and later remyelination in adulthood. Abnormalities in these processes have been associated with behavioral and cognitive dysfunctions and the development of various mental illnesses like schizophrenia. Several studies have implicated oligodendrocyte dysfunction and myelin abnormalities in the disorder, together with altered expression of myelin-related genes such as Olig2, CNP, and NRG1. However, the molecular mechanisms subjacent of these alterations remain elusive. Schizophrenia is a severe, chronic psychiatric disorder affecting more than 23 million individuals worldwide and its symptoms usually appear at the beginning of adulthood. Currently, the major therapeutic strategy for schizophrenia relies on the use of antipsychotics. Despite their widespread use, the effects of antipsychotics on glial cells, especially oligodendrocytes, remain unclear. Thus, in this review we highlight the current knowledge regarding oligodendrocyte dysfunction in schizophrenia, compiling data from (epi)genetic studies and up-to-date models to investigate the role of oligodendrocytes in the disorder. In addition, we examined potential targets currently investigated for the improvement of schizophrenia symptoms. Research in this area has been investigating potential beneficial compounds, including the D-amino acids D-aspartate and D-serine, that act as NMDA receptor agonists, modulating the glutamatergic signaling; the antioxidant N-acetylcysteine, a precursor in the synthesis of glutathione, protecting against the redox imbalance; as well as lithium, an inhibitor of glycogen synthase kinase 3β (GSK3β) signaling, contributing to oligodendrocyte survival and functioning. In conclusion, there is strong evidence linking oligodendrocyte dysfunction to the development of schizophrenia. Hence, a better understanding of oligodendrocyte differentiation, as well as the effects of antipsychotic medication in these cells, could have potential implications for understanding the development of schizophrenia and finding new targets for drug development.

## Introduction

Oligodendrocytes (OLs) are the neuroglial cells responsible for myelin sheath formation in the central nervous system (CNS). The life cycle of these cells consists of a series of coordinated and highly regulated processes, including migration, proliferation, maturation, and myelination of oligodendroglial cells, leading to adequate brain connectivity (1). In fact, the importance of OL development and myelination in neuronal signaling is well established, as evidenced by several behavioral and cognitive functions (2). Moreover, decreased myelination and OL alterations have been seen in patients with schizophrenia (3), associated with cognitive dysfunction (4). Recently, some studies have also proposed that OLs are more vulnerable to energy metabolism dysfunctions, oxidative damage, and other brain injuries (5, 6), leading to the impaired white matter integrity and brain dysconnectivity observed in schizophrenia (7). Nevertheless, the molecular mechanisms underlying these alterations remain elusive.

Schizophrenia is considered a multifactorial psychiatric disorder that affects more than 23 million individuals worldwide. This disorder is characterized by positive symptoms (delusions and hallucinations), negative symptoms (impaired motivation and social withdrawal), and cognitive impairment. Moreover, environmental and genetic factors, as well as neurodevelopmental dysfunction, play a role in the pathogenesis of schizophrenia (8, 9). The pathophysiology of the disorder includes molecular abnormalities in the nervous, immune, metabolic, and endocrine systems. Several hypotheses have been proposed to describe the etiology of schizophrenia, among which are the well-known dopaminergic and glutamatergic ones.

The dopaminergic hypothesis was developed during the 1970s in association with the discovery of antipsychotics' action on the blockade of D2 dopaminergic receptors (10). Over time, compelling evidence of altered dopaminergic transmission, involving D1 and D2 receptors, was shown in different brain areas linked to the development of positive symptoms and cognitive impairment in schizophrenia (11).

As for the glutamatergic hypothesis, antagonists of N-methyl-D-aspartate (NMDA) receptors mimic alterations and worsen symptoms observed in patients with schizophrenia such as impaired memory, hallucinations, delusions, and agitation (12, 13). This led to the postulation of a glutamatergic hypofunction in the disorder (14). Moreover, altered expressions of NMDA receptors and enzymes involved in glutamate metabolism have been found in patients with schizophrenia (15, 16).

Although there is evidence pointing toward these two aforementioned hypotheses, several lines of evidence also suggest the involvement of white matter abnormalities and neural dysconnectivity in the pathogenesis of schizophrenia (7). For instance, brain imaging and *postmortem* studies have demonstrated alterations in the myelination of cortical areas and white matter tracts, as well as a reduced number of OLs in patients with schizophrenia (3, 17). During neurodevelopment, dynamic and timely regulated changes in gray/white matter ratios appear to be crucial for adequate brain function (18, 19). During these processes, myelination occurs alongside neuronal development and ensures the brain's functional synchrony. Schizophrenia has been proposed to arise in part from faulty brain connectivity (20) and mounting evidence supports the notion of OL/myelination dysfunction as its underlying cause [reviewed in (21, 22)]. Despite the significant progress achieved in the comprehension of this multifactorial disorder in the past decades, the etiology of schizophrenia still remains unclear (23).

Currently, the main therapeutic strategy for the disorder relies on the use of antipsychotics. These medications mainly attenuate the positive symptoms, though atypical antipsychotics can induce slight improvements in cognitive and negative traits (24). Unfortunately, these same medications are also well-known for their severe side effects, including metabolic syndrome and extrapyramidal effects, often leading to treatment discontinuation. In an attempt to change this situation, it is important to investigate the molecular mechanisms involved in antipsychotics' effects beyond neurons and the classical components of neurotransmission. OLs are one of the structures to be investigated to increase the understanding of the development of schizophrenia and find new targets for novel therapies.

In this review, we first describe the differentiation of OLs and myelination, followed by the pathways and signals involved in these processes. Secondly, we discuss how dysregulations in OL function may be linked with schizophrenia, as well as the main pathways and genes involved with OLs and this disorder. We also review current approaches being used to investigate the role of OL dysfunction in schizophrenia. Finally, we provide an overview of the effects of antipsychotics on the proliferation and differentiation of OLs, and its potential for the discovery of novel therapeutic targets, suggesting new avenues for future research in the field.

## OL Development and Myelination

The oligodendroglial cells are characterized as being cells that progressively mature from OL progenitor cells (OPCs) to myelinating cells through a series of coordinated and highly regulated programs of migration, proliferation, and differentiation, up to the point at which OLs become myelinating cells (1, 25). OPCs originate from neural stem cells (NSCs) that initially differentiate into neural progenitor cells (NPCs) in the subventricular zone (SVZ) (26). NPCs then give rise to OPCs, which migrate radially out of the SVZ to cortical and white matter areas (27, 28).

The maturation of OLs is controlled by transcription factors, and cell-extrinsic molecules, including small metabolic molecules, chemokines, growth factors, neurotransmitters, and hormones that act at defined time points during embryonic and postnatal development (29). Moreover, chromatin remodeling and epigenetic regulation, including methylation and histone modifications, are associated with OL development (30). During the initial stages of differentiation, histone deacetylation promotes chromatin compaction, ensuring that the transcription of non-OL genes is inhibited, followed by the induction of activators of OL cell fate (31, 32). In addition, DNA methylation plays a significant role in OPC expansion and regulates processes required for *de novo* myelination and remyelination, such as RNA splicing and protein synthesis (33, 34).

During maturation, cells committed to an OL lineage go through a series of specific phenotypic stages, including OPCs, pre-OLs, immature (or pre-myelinating) OLs and mature (or myelinating) OLs (1). These differentiation steps can be distinguished by migratory cell capacity, morphological complexity, and sequential expression of stage-specific markers in response to differentiation signals (1).

Initially, OPCs are proliferative cells that migrate and give rise to pre-OLs. These cells project multipolar processes and start to express OPC markers, such as O4, neuron-glial antigen 2 (NG2), A2B5, and OL lineage transcription factor 2 (Olig2) (35, 36). In turn, pre-OLs differentiate into immature OLs, post-mitotic cells that present long ramified branches (36). Immature OLs begin to express 2′, 3′-cyclic-nucleotide-3-phosphodiesterase (CNP) (37) and galactocerebroside C (GalC) while maintaining O4 expression and losing expression of NG2 and A2B5 markers (38). Throughout differentiation, OLs lose their ability to migrate and proliferate, acquiring a more complex morphology (39). Mature OLs extend axon-wrapping membranes and express myelin-associated proteins, among which are proteolipid protein (PLP), myelin basic protein (MBP), myelin OL glycoprotein (MOG), and myelin-associated glycoprotein (MAG) (40, 41).

Structurally, the myelin sheath is a multilayered, tightly packed membrane wrapped around selected nerve axons. It consists mainly of lipids (70%–80% dry weight—cholesterol and ethanolamine plasmalogen are the most predominant) and proteins (∼20%–30% content) (42). The myelin sheath provides essential conditions for the conduction of saltatory nerve impulses, such as electrical resistance and reduced capacitance. Axonal myelination by OLs is a remarkable example of dynamic cell membrane specialization and remodeling, involving cytoskeletal rearrangements, along with cell–cell and cell–extracellular matrix interactions (43). Cytoplasmic compaction of the myelin lamellae provides the physical stability necessary for membrane wrapping, leading to cytoplasmic extrusion and adhesion of multiple layers of myelin (43). Therefore, during differentiation, the expression of negatively charged sialic acids by OLs is reduced, thereby decreasing electrostatic repulsion forces, which contributes to the adhesiveness and compaction of the myelin bilayer (44).

MBP and PLP are the two principal proteins present in compact myelin, increasing the adhesiveness of the myelin membrane, and contributing to myelin stability (44, 45). In contrast, CNP prevents myelin compaction through the rearrangement of the cytoskeleton (46). Thus, the interaction between MBP, PLP, and CNP is essential to avoid altered myelin compaction and to the maintenance of myelin stabilization (for review see (43)]. Besides those molecules, there are various biochemical pathways and signaling compounds released by other glial and neuronal cells that also contribute to the regulation of various stages of OPC differentiation and OL myelination, resulting in a complex and integrated network.

### OL Development and Myelination: Signals and Pathways

In the brain, oligodendrogenesis and myelination require the activation of several signaling pathways, as well as transcription factors, noncoding RNAs, that act on the regulation of the different stages of OLs differentiation (47). Moreover, these processes are influenced by the interaction of astrocytes (48), microglia (49), and neurons with OLs and their precursors (50).

Regarding the role of glial cells in these interactions, astrocytes provide cholesterol precursors and nutrients for myelin sheath synthesis, in addition to chemokines and growth factors that influence OL survival, proliferation, migration, and maturation (48, 51). Similarly, microglia regulate oligodendrogenesis by releasing growth and attraction factors, including platelet-derived growth factor (PDGF), insulin-like growth factor-1 (IGF-1) (52), and hepatocyte growth factor (HGF) (53). Moreover, neurotrophic factors such as brain-derived neurotrophic factor (BDNF) and nerve growth factor (NGF) are known to guide axonal myelination (54, 55). Certain neurotransmitters and hormones can also stimulate oligodendroglial cells by mechanisms not yet completely understood (56).

Several pathways regulate OL differentiation and myelination in the CNS. The phosphatidylinositol-3-phosphate kinase (PI3K)/Akt/mTOR pathway, for example, regulates the initiation of myelination in the CNS through mTORC1 signaling (57). In conjunction, glycogen synthase kinase 3β (GSK3β) signaling regulates OL differentiation (58). Its inhibition induces OL proliferation and differentiation, as well as remyelination in mouse models of demyelination (58). Moreover, *in vivo* studies with mice overexpressing Akt also revealed an essential role of this pathway in inducing myelination rather than the proliferation of OLs (59).

Moreover, extracellular signal-regulated kinases-1 and -2 (ERK1/2), the downstream mediators of the mitogen-activated protein kinases (MAPKs) pathway, regulate myelin growth and maintain the integrity of myelinated axons (60). These MEK/ERK1/2-MAPK-mediated functions are largely independent of mTORC1 (61). Nonetheless, both pathways induce myelin sheath growth mediated by signaling responses involving mTORC1 (61). Therefore, PI3K/Akt/mTOR and MEK/ERK1/2-MAPK signaling pathways act both independently and in conjunction to temporally regulate OL differentiation and myelination (61). Disruptions in the finely tuned regulatory processes of OL development and myelination could result in white matter disturbances linked to the etiology of several mental illnesses, including schizophrenia. These pathways are also associated with the treatment of this disorder, holding promising findings as potential targets for novel therapeutic strategies.

## OLs and Myelin Impairment in Schizophrenia

Disruptions during brain development caused by (epi)genetic and environmental factors can result in brain damage which could interfere in its ability to maintain normal communication across functional neural networks [for review see (62)]. Myelination is essential for adequate brain function and must occur alongside neuronal development and is only completed within the third decade of life. Disruptions in this process have been linked to the development of schizophrenia [for review see (63)].

The hypothesis that OLs play a major role in schizophrenia is corroborated by genome-wide expression analysis revealing altered expression of myelination-related genes in patients with schizophrenia and white matter pathologies that impair brain functional synchrony thereby resulting in schizophrenic symptoms (64–66).

In addition, structural changes have been reported in white matter tracts in the brains of patients with the disorder (67, 68). The anterior hippocampus plays an important role in the regulation of emotions and deficits in this area have been linked to schizophrenia. Studies have shown a decreased number of OLs in the hippocampus causing a disturbance in the neurocircuitry between the hippocampus and hypothalamus leading to cognitive deficits in patients with schizophrenia (69, 70). Furthermore, myelination deficits and dysfunctional synapses observed in the disorder can be correlated to the dysfunction of neuronal and glial function (71–73), along with cognitive impairment, including deficits in episodic and working memory (74) and in visual processing (75). Moreover, disruptions in OL integrity such as altered morphology and distribution (3), decreased density (76), and altered expression of myelination-related genes and proteins have been reported in patients with schizophrenia (71, 77, 78).

Epigenetics has also been suggested as an etiopathogenic factor for complex mental disorders, including schizophrenia (79). Aberrant epigenetic signatures, like hypermethylation, may be caused by well-known risk factors for schizophrenia during neurodevelopment such as drug abuse during pregnancy, in utero viral infections and hypoxia (80, 81). Recent advances in sequencing technologies have allowed researchers to identify associations between schizophrenia and epigenetic modulations *via* DNA methylation and histone modifications (82–86). These have been shown to affect genes well-known for their role in OL lineage development (87), however, the majority of differential epigenetic signatures between OLs of control and schizophrenia brain samples have been found in non-coding regions (88).

Further insight into the pathology of schizophrenia and its connection with OL abnormalities has come from comparative proteomic analyses that reveal differentially expressed proteins and disturbances in biochemical pathways, including energy metabolism, oxidative stress and OL-related functions (78, 89). This is in line with an earlier brain imaging study that demonstrated decreased glucose metabolic rates in different thalamic nuclei in patients with schizophrenia (90). Nevertheless, schizophrenia pathophysiology also includes genetic factors. For this reason, several OL and myelin-related genes are being investigated in *postmortem*, *in vivo* and *in vitro* approaches to understand the contribution of genetic factors to OL development (73).

### OL- and Myelin-Related Genes in Schizophrenia

Several (epi)genetic alterations, including risk variants and polymorphisms, have been demonstrated to play an important role in the etiology of schizophrenia (91, 92). Moreover, there are several promising candidate susceptibility genes associated with OL dysfunctions, such as Neuregulin 1 (NRG1)–ERBB4 signaling, Nogo-A/Nogo Receptor, Disrupted-in-schizophrenia 1 (DISC1), and other myelin-related genes (71, 93, 94). In an attempt to unravel the mechanisms involved with OL dysfunction in schizophrenia, OL- and myelin-related genes, as well as its regulatory signaling pathways are being investigated to understand how (epi)genetic and molecular changes lead to schizophrenia. Here we review the data available concerning those candidate myelin-related genes in the disorder.

#### NRG1–ERBB4 Signaling

NRG1 and its receptor ERBB4 are essential for neuronal, astroglial, and OL development, playing an important role in the survival and differentiation of these glial cells (95, 96). NRG1 is expressed at CNS synapses and activates neurotransmitter receptors, such as the glutamatergic ones (94). This signaling pathway has been linked to schizophrenia as a potential genetic risk factor in genetic and *in vivo* studies (94, 97). NRG1 mutant mice have been shown to present altered behavior, including hyperactivity and a deficit in prepulse inhibition (PPI), which can be attenuated by treatment with the antipsychotic clozapine (CLZ) (98). The mice that were hypomorphic for NRG1 also had fewer functional NMDA receptors in the brain, a feature observed in schizophrenia patients (94). Moreover, NRG1 risk variants linked to schizophrenia have also been associated with reduced white matter density and integrity (99, 100). Regarding its functions in the CNS, the NRG1/ERBB4 signaling pathway operates in several aspects of neurodevelopment, including neural migration, gliogenesis, and modulation of neurotransmission [for review see (101)]. In addition, NRG1 is also involved with OL survival, migration, and myelination (96, 102, 103). Thus, the oligodendroglial association between this pathway and schizophrenia may be another potential alteration linking NRG1 to the development of the disorder (73).

#### Nogo-A and Nogo Receptor

The Nogo-A protein and its receptor (NGR) play an important role in the CNS through repairing processes, restricting axonal growth and plasticity (104, 105). This protein is extensively expressed in OLs, acting in the differentiation of these cells (106, 107). *In vivo*, mutant mice lacking Nogo-A exhibited impaired myelin synthesis and delayed differentiation (107). Moreover, Nogo-A knockout mice showed deficiencies in PPI and latent inhibition, as well as hyperactivity and other behavioral traits that resemble alterations observed in patients with schizophrenia (108). The induction of Nogo-A dysfunction in adult mice failed to reproduce the alterations observed in the mutant mice (108). Hence, Nogo-A dysfunction during neurodevelopment may affect neuronal and glial differentiation (107, 109). Altered expression and polymorphisms of the RTN4 (Reticulon 4) gene, which codes for Nogo-A, have been associated with psychiatric disorders like schizophrenia (110). Thus, alterations involving Nogo-A, NGR and other associated pathways, could contribute to some abnormalities observed in the disorder, such as abnormal neuronal organization and connectivity. Further research is needed to help underline the molecular mechanisms linking Nogo-A and NGR to the pathophysiology of schizophrenia [for review see (111)].

#### Disrupted-in-Schizophrenia 1

DISC1 is a gene disrupted by a chromosomal translocation that has been linked to psychiatric conditions, including schizophrenia, but its association with the disorder remains controversial (93, 112). Studies using *in vivo* models with mutant, truncated DISC1 observed altered OL differentiation and proliferation and alterations in OPC marker levels (113, 114). Investigation of altered gene expression profiles linked to DISC1 has found an association with altered levels of OL markers, such as CNP, MAG, and PLP (114), which contribute to altered white matter functioning and integrity (115). Moreover, *postmortem* studies have revealed an increased expression of DISC1 protein in OLs in frontal-parietal white matter region in paranoid schizophrenia (116). Thus, DISC1-related polymorphisms may contribute to neurodevelopmental alterations, as well as changes in white matter integrity, participating in the etiology of schizophrenia (115).

#### Olig2

Olig2 encodes for a transcription factor essential for several aspects of neurodevelopment, including oligodendrogenesis, the formation of motor neurons, and OL differentiation, especially in the early stages of OPC differentiation (117, 118). In *postmortem* studies, mRNA levels of Olig2 have been found reduced in patients with schizophrenia compared to healthy individuals (71, 119). Studies have described the expression of Olig genes in white matter and during remyelination in a demyelination model (120, 121). Other genes involved with myelination have been correlated with Olig2 expression, including ERBB4 and CNP, which may indicate potential shared pathways and interactions among these genes (122).

#### Other Myelin-Related Genes

Aside from the genes discussed above, there are other genes that have been found to be differentially expressed in patients with schizophrenia which are related to OLs. MAG, CNP, PLP1, and MBP were found reduced in mRNA samples from patients with schizophrenia in *postmortem* studies (71, 123). Genetic and neuroimaging data have shown that the MAG, Olig2, and CNP genes can influence white matter integrity and cognitive performance in schizophrenia patients (124). As previously mentioned, CNP is a marker for OLs during neurodevelopment and is also maintained in mature OLs (38, 125). It has been proposed that the inhibition of CNP in OLs can affect an essential glial trophic support mechanism of axons (126).

Another genetic study evaluated the mRNA expression of Quaking I (QKI), finding two splice variants to be downregulated in patients with schizophrenia (127, 128). QKI is an RNA-binding protein important for the development of glial cells and myelin synthesis (129). In an *in vivo* model, mutant mice (qk^v^) with a deletion in the 5′ regulatory region of the QKI gene presented severe demyelination, altered myelin structure, and reduced lipid content (130, 131). This gene is involved in the regulation of other genes essential for OL differentiation, such as PLP1, MAG, and MBP (132, 133).

Moreover, the Fasciculation and Elongation Protein Zeta-1 (FEZ1) is another myelin-related gene associated with risk for schizophrenia. FEZ1 is a gene involved with OL differentiation and it is known to interact with other schizophrenia-related genes, including DISC1 and QKI, mentioned above (134). FEZ1 has also been found reduced in the hippocampus of patients with schizophrenia and its expression has been associated with disrupted regulation of transcription factors and altered (epi)genetic regulation, including histone acetylation and chromatin remodeling (134, 135).

Changes in (epi)genetic regulation in myelin-related genes have been associated with impaired OL function and development (30). Inhibition of methylation reduces the number of OLs, evidencing the vulnerability of these glial cells to (epi)genetic alterations (136). Moreover, methylome analysis of different cell types isolated from brain tissue of patients with schizophrenia and controls revealed altered methylation associated with OLs (88). For instance, SOX10, a well-known marker of OL development has been shown to be hypermethylated in schizophrenia patients (87); this methylation status may be correlated to its downregulation and OL dysfunction in the disorder.

Therefore, the (epi)genetic components partially explain the etiology of schizophrenia and also support the notion that oligodendroglial abnormalities are among the primary deficits in the disorder (73). However, the individual and collective roles of all these genes remain unknown, making it still somewhat controversial in relation to schizophrenia. Environmental factors have also been associated with the etiology of the disorder, including complications during pregnancy and prenatal maternal infections (137, 138). These scenarios may lead to hypoxic damage and an inflammatory state, which have been linked to gray and white matter dysfunctions (139, 140).

Together, genetic susceptibility and certain environmental conditions may contribute to the development of (epi)genetic dysregulations. Hence, the investigation of the molecular mechanisms behind these different risk factors is essential for the comprehension of the etiology of schizophrenia. Data provided by brain imaging studies, genetic (71, 141) and *postmortem* studies (142, 143), in addition to animal models (94, 98) have been crucial to unravel these mechanisms as well as to understand the role of OL dysfunction in schizophrenia.

## Models of OL and Myelin-Related Disorders

To better understand the molecular changes in OLs and myelination dysfunctions, studies have used different *in vitro* and *in vivo* models to investigate abnormalities observed in OPC differentiation and myelination processes. Aiming to bring together the interactions between genetic and environmental factors, experiments using induced pluripotent stem cells (iPSCs) derived from patients with schizophrenia are being developed to generate OPCs and mature OLs. These tools can help us comprehend the association between OL abnormalities and the development of the disorder as well as aid in screenings for novel therapeutics (144, 145). In addition to iPSCs, mouse primary OPC cultures and oligodendroglial cell lines (MO3.13 and HOG) have also been used to understand the biological processes underlying OL differentiation into myelinating OLs (146–148).

### Human-Induced Pluripotent Stem Cells-Derived OPCs

For neurobiological studies related to the CNS, patient-derived human iPSCs (hiPSCs) are able to be reprogrammed into neuronal and glial cell types, including OPCs and OLs, offering potential tools to elucidate the molecular and cellular mechanisms behind the etiology of schizophrenia (149). One of the main advantages of iPSCs is the conservation of the original genotype and endophenotype from schizophrenia patients (150, 151). Thus far, studies of psychiatric disorders using patient-derived iPSCs have focused on neuronal differentiation (152–154) and have demonstrated decreased neuronal connectivity, increased oxidative stress and mitochondrial dysfunction and, consequently, an impairment in NPC differentiation and neuronal maturation (151, 155).

Recently, studies have started looking toward OPCs derived from iPSCs in neurodegenerative and psychiatric diseases to understand specific aspects of the myelination process and to investigate how dysfunctional neuron–OL interactions manifest in schizophrenia (146, 156). However, currently available protocols for the differentiation of iPSCs into OPC are still very limited and are not able to induce differentiation into a mature myelinating OL. Wang et al. (2013) developed a protocol to produce myelinogenic OLs from skin-derived hiPSCs and showed the potential therapeutic of hiPSC-derived OPCs as a treatment for disorders of myelin loss (157). In the context of schizophrenia, a protocol used to generate hiPSC-derived OPCs from patients confirmed that OL dysfunction and anomalies were similar to the alterations in white matter integrity observed in magnetic resonance imaging studies from patients with schizophrenia. Thus, both models confirm OL abnormalities in schizophrenia, linking OL dysfunction with a decreased number of OPCs (145).

#### Primary OPC Culture and Oligodendroglial Cells Lines

Primary glial cell cultures from mice and rats are the most common and well-established *in vitro* model used for the study of diseases that affect the CNS and allow the study of OPC differentiation and OL function at the cellular and molecular levels (158, 159). One advantage of primary OPC culture is the availability of protocols for the isolation of OLs, microglia and astrocytes from mixed glial cultures. Such protocols are an economical, simple and reproducible way to obtain oligodendroglial cells with high purity and high efficiency, besides requiring a reduced number of animals (160, 161).

Following the pathway of practical, fast and economical *in vitro* models, the oligodendroglial cells lines MO3.13 and HOG have also been used to analyze OL differentiation and abnormalities (162). MO3.13 is an immortal human-human hybrid cell line, created by fusing rhabdomyosarcoma cells with adult OLs (163). The HOG cell line is directly derived from a human glioma that expresses OL markers MBP and CNPase but does not express glial markers, such as GFAP and glutamine synthase (164). MO3.13, however, expresses GFAP and also exhibits GalC and CNPase immunoreactivity (163). Upon differentiation, MO3.13 cells express MBP and PLP, which are markers for mature OLs (163, 165). Both cell lines have been employed as models to study the cellular neurobiology in OL-linked diseases, such as multiple sclerosis (166) and schizophrenia (5, 167). Unfortunately, protocols to differentiate HOG and MO3.13 into mature OLs vary greatly and these cell lines do not reach a myelinating state, which jeopardizes a more comprehensive study of myelination process using these models (148).

To study the neurobiological alterations in schizophrenia, including the glutamatergic dysfunction in OL, the MO3.13 cell line was treated with MK-801, a glutamatergic NMDA receptor antagonist that mimics the hypofunction in glutamatergic signaling seen in schizophrenia patients (165). The results exhibit differentially expressed proteins related to energy metabolism and glycolysis (5), including proteins previously associated with schizophrenia (165). Moreover, MK-801-treated OLs present more severe alterations compared to neuronal and astrocyte cell lineages, causing OLs to exhibit increased vulnerability to NMDA and glutamatergic dysfunction (5). Given the OLs' high energy demand to promote axon myelination, these metabolic disturbances could lead to white matter dysfunction and, consequently, altered neuronal connectivity, thereby playing a major role in the etiology of schizophrenia (168, 169).

Despite partially representing disturbances observed in schizophrenia and comprising critical tools in understanding OLs and white matter dysfunctions in schizophrenia, the use of isolated OL cell line cultures cannot reproduce complex physiological processes such as the communication between OLs and neurons, which is an essential factor for *in vivo* myelination and remyelination. In this context, the development of neuronal and glial co-culture systems, brain organoids, and organotypic slice cultures come much closer to the biological conditions present in a physiological environment (73, 151) Furthermore, a more robust way to study cell–cell interactions in the pathogenesis of schizophrenia and to better understand how OL-neuron interactions contribute to abnormal myelination, is by using the *in vivo* models of demyelinating diseases and remyelination processes.

#### *In Vivo* and *In Vitro* Strategies Using Cuprizone-Induced Demyelination

The cuprizone (CPZ; bis-cyclohexanone oxaldihydrazone) model is a common animal model used to investigate demyelination-associated diseases such as multiple sclerosis and schizophrenia (170). CPZ is a copper chelator that impairs the activity of copper enzymes (cytochrome oxidase and monoamine oxidases), developing reversible, region-specific OL loss and demyelination, as well as stimulating microgliosis and astrogliosis (171–173). The CPZ hypothesis proposes that perturbation in energy metabolism contributes to OL apoptosis and induces demyelination [for review see (170)]. Furthermore, CPZ reduces the expression of myelin-related genes and proteins, such as MBP, MOG, PLP, and CNP in animals' corpus callosum (174), consequently inducing brain demyelination, myelin disruption, and OL loss (170, 174, 175). This animal model manifests symptoms similar to those observed in schizophrenia patients, such as deficits in working memory (176, 177) and in PPI of the acoustic startle response, less social interaction, and higher dopamine in the prefrontal cortex (PFC) (175), showing that OL dysfunction and abnormal myelination triggers schizophrenia-like symptoms.

The main CPZ-toxic target are mature OLs. CPZ also has a toxic effect in OLs cultured cells, causing a direct disruption of OL function. For instance, Taraboletti (2017) showed that CPZ induces cell death in the MO3.13 cell line through changes in oligodendroglial energy metabolism (178). CPZ is not able to exert toxic effects on microglia, astrocytes, and SH-SY5Y cells, showing a selective effect in OLs. Moreover, CPZ inhibits the differentiation and maturation of cultured OPCs and decreases the levels of CNP and MBP in mature OLs (179). These results highlight the selective demyelination induced by CPZ *in vivo*, showing that the CPZ model may be a potential experimental tool for assessing molecules related to demyelination and remyelination (170, 180).

Currently, it is unknown whether OL dysfunction consists of a primary or a secondary cause of neuronal and synaptic abnormalities in schizophrenia. Thus, it is important to study cell–cell interactions in the schizophrenia pathologic process. All the models described above partially represent OL and myelination disturbances observed in schizophrenia, comprising critical tools in understanding OLs and white matter dysfunction in this disorder. However, schizophrenia is a multifactorial psychiatric disorder, involving environmental and genetic factors, as well as neurodevelopmental dysfunction (8, 9). For this reason, developing a model that links genetic and environmental elements with cellular and molecular alterations associated with positive and negative symptoms, and cognitive impairment is still a challenge.

Molecular pathogenesis research using iPSC-related technologies, such as three-dimensional (3D) culture models and organoids, offers new insight to recapitulate the complexity of the human CNS. Novel findings derived from these models, associated with clinical phenotype in postmortem, *in vivo*, *in vitro* approaches and findings from brain magnetic resonance imaging and genomic studies, will be increasingly important for a better understanding of the processes involving OL dysfunction in schizophrenia (151).

## Antipsychotics and OLs

Currently, the major therapeutic strategy for schizophrenia relies on the use of antipsychotic drugs. Antipsychotics are high-affinity antagonists of dopamine D2 receptors and are categorized into two classes: typical, or first-generation and atypical, or second generation. The typical antipsychotics, such as haloperidol (HAL), act mainly through dopamine D2 antagonism, thereby improving positive symptoms (10, 181). These antipsychotics induce severe extrapyramidal side effects and have a high incidence of nonresponders (182). In contrast, atypical antipsychotics, such as CLZ, inhibit dopamine D2 as well as serotonergic receptors (especially 5-HT_2A_ and 5-HT_2C_) (183). They attenuate principally positive symptoms and induce fewer neurological side effects; however, they can lead to metabolic disturbances (184).

Antipsychotics were found to target OL development, reinforcing the role of OLs in the pathophysiology and treatment of schizophrenia (185). *In vitro* and *in vivo* studies have been investigating the effects of atypical and typical antipsychotics on the proliferation and differentiation of glial cells (185–187). The results obtained so far remain controversial; while one study reports that the typical antipsychotic HAL promotes proliferation but not the differentiation of OLs (188) another one confirmed HAL inhibition of OL differentiation but found no effects on OL proliferation (186, 188). Regarding the atypical antipsychotics, olanzapine and quetiapine (QUE) induce OL proliferation, however, only QUE promotes OL differentiation, through ERK1/2 signaling and increases CNP and MBP levels (176).

Recent studies have found that the atypical antipsychotic QUE, administered both before or after CPZ-mediated demyelination, significantly enhances OL regeneration and myelin repair by accelerating the maturation of OPCs (187, 189). This acceleration promotes the survival of OLs, linked to a downregulation of the transcription factor Olig2 in the process of cell maturation (189). Regarding behavioral changes, QUE treatment during the remyelination period improves spatial working memory (189) and cognitive impairments (176) and reverses the deficit in PPI of the acoustic startle response in CPZ-treated mice (175, 177). Antipsychotic treatment (CLZ, QUE, and HAL) in the CPZ model reversed higher dopamine levels in the PFC and lowered spontaneous alternations in Y-maze tests induced by CPZ (175). The study also revealed that, in mice treated with CPZ, the white matter damage in the PFC was reduced after treatment with CLZ or HAL. Moreover, in the caudate–putamen and hippocampus white matter loss was only attenuated by CLZ and QUE (190).

Moreover, in a mouse model of social isolation, animals treated with QUE presented reduced myelin deficits and increased histone methylation in OLs, besides an improvement in social interactions (191). QUE has also been associated with increased methylation of risk factor genes implicated with schizophrenia, providing evidence that epigenetic regulation may be involved in the mechanisms of action of this antipsychotic (192).

Other antipsychotics have also been associated with epigenetic modifications. *In vivo*, OLA induced altered methylation of genes involved with dopaminergic signaling in the hippocampus and cerebellum (193); CLZ was able to reverse epigenetic alterations induced by phencyclidine treatment in mice, improving cognitive dysfunction, memory recognition, and social deficit (194).

Regarding biochemical pathways, it has been shown that antipsychotic effects are associated with cortical and gray/white matter intensity and with lipid metabolism alterations (195, 196). CLZ and HAL can alter OL glucose metabolism, which is deregulated and correlated with abnormal behavior and cognition in schizophrenia patients (196). Moreover, these drugs are able to stimulate the synthesis of cholesterol and fatty acids in glial cells through regulation of gene expression mediated by the sterol regulatory element-binding proteins SREBP-1 and SREBP-2 (197). Glia-derived cholesterol is essential for the formation of myelin and synapses in the CNS (198). Cholesterol metabolism has been implicated in the regulation of many processes, including myelin membrane growth, axon wrapping, and synapse formation. Thus, cholesterol and myelin synthesis are essential for brain physiological homeostasis (199).

Moreover, reduced Akt signaling has been found in the frontal cortex of *postmortem* brain tissue from patients with schizophrenia (200, 201). *In vivo* animal models modulating Akt signaling have also exhibited schizophrenia-like behaviors and abnormalities (202, 203). Another *in vivo* study investigated the effect of HAL on Akt signaling, finding increased phosphorylation of Akt as a potential protective mechanism of the medication (200). PI3K/AKT/mTOR signaling is a well-established pathway related to several biological processes, including protein synthesis and cell growth, also playing an important role in OL survival (59). Proteomic analyses of the human immature oligodendroglial cell line MO3.13 found altered proteins associated with the mTOR signaling pathway after treatment with atypical (risperidone and QUE) and typical antipsychotics (chlorpromazine and HAL) (204). According to this study, these medications resulted in different protein expression levels, revealing potentially distinct effects of those drugs on OLs.

As such, upstream and downstream processes linked with PI3K/Akt/mTOR signaling may not only influence OL functioning but may also be associated with the molecular mechanisms of antipsychotics. One example is Akt and GSK3β signaling, found to be mediated by cyclin-dependent kinase 5 (Cdk5) (205); in this study, the *in vivo* model for Cdk5 knockout resulted in impaired remyelination and reduced phosphorylation levels of Akt and GSK3β, indicating the potential role of Cdk5 in the regulation of both pathways. Reduced phosphorylation of GSK3β inhibits differentiation and myelination of OLs (58). As a result, GSK3β inhibitors are being investigated as potential targets for remyelinating drugs such as lithium, discussed in the following section.

In summary, aberrations in OL development and myelin contribute to the pathogenesis of schizophrenia and drugs that reverse abnormal OL phenotypes may be a promising treatment strategy. OL development is a target of some antipsychotics (185) and understanding the effects of antipsychotics on OLs reinforces the need for future therapeutic intervention studies.

## Novel Potential Therapeutic Targets

In this review, OL maturation and function in the CNS have already been discussed, detailing how these cells may be involved in the pathophysiology of schizophrenia. From *postmortem* brain studies to *in vivo* and *in vitro* models, several approaches were reviewed, all of which have been used to investigate OL dysfunctions in this psychiatric disorder.

Data from these different strategies exhibit alterations in myelin-related genes in patients with schizophrenia. Further investigation of these candidate genes may contribute to the search for possible targets for drug development. Given the association of white matter dysfunction with brain dysconnectivity and the development of positive, negative, and cognitive symptoms observed in schizophrenia (206–208), the investigation of potential promyelination agents and other protective substances during myelination in the CNS could provide strategies to prevent the damage caused by altered myelination and other OL dysfunctions.

As for typical and atypical antipsychotics, since they are the main therapeutic strategy currently used for the treatment of schizophrenia, a better understanding of their effects on the proliferation and differentiation of OLs is crucial. Their molecular mechanisms and effects on the white matter remain elusive and more data are necessary to determine any protective and negative effects of those drugs after short- and long-term treatment, especially related to white matter integrity.

In regard to glutamatergic dysfunction, altered signaling and receptors dysfunction have been described in schizophrenia (14). Glial cells are especially vulnerable to glutamate excitotoxicity (209), resulting in impaired OL functioning and white matter lesions (210). Therefore, the investigation of compounds able to prevent this excitotoxicity could provide protective effects, especially to attenuate white matter injury. One example is memantine, a non-competitive NMDA antagonist with a low binding affinity that acts by partially blocking this receptor, preventing glutamate toxicity (211, 212).

Other NMDA modulatory compounds include NMDA receptor agonists, such as D-amino acids: D-aspartate and D-serine. D-aspartate also acts as an NMDAr precursor and is essential in several processes in the CNS, including cognitive function (213, 214). Regarding its role in OLs, D-aspartate contributes to remyelination and is also associated with OL maturation and differentiation (215). D-serine is an endogenous ligand for the glycine site of NMDA receptors. Together with glutamatergic signaling, D-serine was found to contribute to the induction and maintenance of long-term potentiation in synaptic plasticity (216). Clinical trials showed that the administration of D-serine alleviated positive, cognitive, and negative symptoms in schizophrenia patients (217).

Given the negative regulation of GSK3β signaling in OLs, GSK3β inhibitors also present promising results for OL differentiation and survival (58). Lithium is one such inhibitor and is a well-known mood stabilizer employed in bipolar disorder treatment. It exhibits neuroprotective properties, promoting increased expression of myelin-related genes and OL remyelination (218, 219). Lithium was also found to improve symptoms involving emotional withdrawal, motor activity, disorganized thoughts, and positive symptoms in patients with schizophrenia (220).

Altered energy metabolism has also been reported in schizophrenia, leading to an impaired redox balance (78). OPCs are more sensitive to oxidative stress and have lower glutathione (GSH) levels compared to other cells like neurons and astrocytes (168). The tripeptide GSH is an essential intracellular antioxidant also found at reduced levels in patients with schizophrenia (221). It has been suggested that antioxidants such as N-acetylcysteine, which acts as a precursor of cysteine in GSH synthesis, could be an adjunctive strategy in the treatment of psychiatric disorders (222).

**Figure 1:**
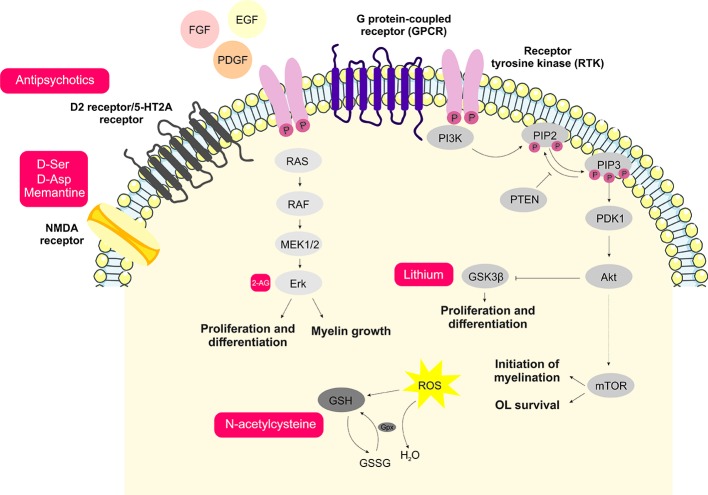
Schematic representation of the main signaling processes and potential novel treatment targets involved with oligodendrocyte dysfunction in schizophrenia. Antipsychotics acting via inhibition of D2 and 5-HT2A receptors are the main therapeutic strategy currently used in schizophrenia treatment. D-amino acids and memantine are shown here as potential targets aiming for improvements in the glutamatergic signaling dysfunction. Endocannabinoids and the GSK3β inhibitor, lithium, contribute to the MEK/ERK1/2-MAPK and PI3K/Akt/mTOR signaling pathways, which are important for oligodendrocyte proliferation and differentiation. Moreover, aiming to attenuate oxidative damage, antioxidants such as N-acetylcysteine may consist of potential therapeutic agents.

Another signaling process that can become a target for drug discovery studies in OLs is the endocannabinoid system. This system acts in the survival, proliferation, migration, and differentiation of OLs (223). OPCs are known to produce the endocannabinoid arachidonoylglycerol (2-AG), consequently stimulating the ERK signaling pathway, inducing OL differentiation and MBP production (224). Cannabidiol (CBD) is a phytocannabinoid that has presented beneficial effects on OLs, protecting OPCs against inflammation-induced damage and oxidative and endoplasmic reticulum stress (225). All potential targets discussed above are summarized in **Figure 1**.

## Conclusion

Due to the high complexity of and interaction between, (epi)genetic and environmental factors linked to the development of schizophrenia symptoms, several pathways, along with white and gray matter dysfunction, have been implicated in the disorder. To date, investigations have focused on neuronal cells and, more recently, some studies have been shifting their focus toward the role of glial cells in schizophrenia. Combined data from different *in vivo* and *in vitro* models, as well as brain imaging and postmortem studies, provide evidence for white matter dysfunction and dysconnectivity, both observed in schizophrenia. However, the molecular mechanisms underlying these alterations remain unclear.

Thus, continued research in the field could provide further knowledge regarding the role of OLs in the pathophysiology of the disorder, as well as the effects of antipsychotic medication on these cells. This, in turn, would contribute to the discovery of novel targets and the development of new approaches targeting cognitive and negative symptoms, designing adjunctive treatments alongside antipsychotics.

## Nomenclature

BDNF, brain-derived neurotrophic factor; CBD, cannabidiol; CLZ, clozapine; CNP, 2′,3′-cyclic-nucleotide-3-phosphodiesterase; CNS, central nervous system; CPZ, cuprizone; DISC1, disrupted-in-schizophrenia 1; ERK1/2, signal-regulated kinases-1 and -2; GalC, galactocerebroside C; GSH, glutathione; GSK3β, glycogen synthase kinase 3β; HAL, haloperidol; IGF-1, insulin-like growth factor 1; MAG, myelin-associated glycoprotein; MAPKs, mitogen-activated protein kinases; MBP, myelin basic protein; MOG, myelin oligodendrocyte glycoprotein; mTOR, mammalian target of rapamycin; NG2, neuron-glial antigen 2; NGR, Nogo Receptor; NGF, nerve growth factor; NMDA, N-methyl-D-aspartate; NPC, neural progenitor cells; NRG1, Neuregulin 1; NSC, neural stem cells; Olig2, oligodendrocyte lineage transcription factor 2; OL, oligodendrocyte; OPCs, oligodendrocyte progenitor cells; PFC, prefrontal cortex; PI3K, phosphoinositide 3-kinase; PLP, proteolipid protein; PPI, prepulse inhibition; QKI, Quaking I; QUE, quetiapine; Rtn4, Reticulon 4; SVZ, subventricular zone.

## Author Contributions

DG-J conceived the study, designed, and wrote the manuscript. AA helped with data interpretation and manuscript revision. GS and AF conceived the study and assisted with the manuscript draft. CB-T conceived the study and designed the figure. FC and DM-d-S conceived the study, supervised the process, and finalized the manuscript. All authors approved the final version of the manuscript.

## Funding

DG-J, AF, GS, CB-T, FC, and DM-d-S are supported by the São Paulo Research Foundation (FAPESP, grant numbers 2018/25439-9; 2017/25588-1; 2018/03673-0; 2018/10362-0, 2017/25055-3; 2019/22398-2). AA and FC are supported by the Coordination for the Improvement of Higher Level Personnel (CAPES/BRAZIL, grant number 465412/2014-9—INBioN).

## Conflict of Interest

The authors declare that the research was conducted in the absence of any commercial or financial relationships that could be construed as a potential conflict of interest.
